# A p.N92K variant of the GTPase RAC3 disrupts cortical neuron migration and axon elongation

**DOI:** 10.1016/j.jbc.2025.108346

**Published:** 2025-02-25

**Authors:** Ryota Sugawara, Keisuke Hamada, Hidenori Ito, Marcello Scala, Hiroshi Ueda, Hidenori Tabata, Kazuhiro Ogata, Koh-ichi Nagata

**Affiliations:** 1 Department of Molecular Neurobiology, Institute for Developmental Research, Aichi Developmental Disability Center, Kasugai, Japan; 2 United Graduate School of Drug Discovery and Medical Information Sciences, Gifu University, Gifu, Japan; 3 Department of Biochemistry, Yokohama City University Graduate School of Medicine, Yokohama, Japan; 4 Department of Neurosciences, Rehabilitation, Ophthalmology, Genetics, Maternal and Child Health, University of Genoa, Genoa, Italy; 5 Unit of Medical Genetics, IRCCS Giannina Gaslini Institute, Genova, Italy; 6 Center for One Medicine Innovative Translational Research (COMIT), Gifu University, Gifu, Japan; 7 Department of Neurochemistry, Nagoya University Graduate School of Medicine, Nagoya, Japan

**Keywords:** *RAC3*, small GTPase, brain development, structure, axon guidance, neuronal migration, pathogenic variant

## Abstract

*RAC3* encodes a small GTPase of the Rho family, crucial for actin cytoskeleton organization and signaling pathways. *De novo* deleterious variants in *RAC3* cause neurodevelopmental disorder with structural brain anomalies and dysmorphic facies (NEDBAF). Disease-causing variants thus far reported are thought to impact key conserved regions within RAC3, such as the P-loop, switch I/II, and G boxes, which are essential for the interaction with regulatory proteins and effectors. Recently, however, a novel variant, c.276T > A, p.N92K, was identified in a prenatal case with complex brain malformations. This variant, located outside the core functional regions, represents a unique class of RAC3 pathogenic mutations. We investigated the variant's effects using *in vitro*, *in silico*, and *in vivo* approaches. Overexpression of RAC3-N92K in primary hippocampal neurons impaired differentiation, leading to round cell shape with lamellipodia, suggesting that RAC3-N92K is active. Biochemical studies showed that RAC3-N92K is (1) resistant to GAP-mediated inactivation, (2) responsive to GEF activation, and (3) capable of interacting with RAC effectors PAK1 and MLK2, as well as Rho-kinase 1, activating gene expression through SRF, NFκB, and AP1 pathways. Structural analyses suggest that N92K disrupts GAP interactions but preserves interactions with GEF, PAK1, and MLK2. *In vivo*, RAC3-N92K expression in embryonic mouse cortical neurons led to migration defects and periventricular clustering during corticogenesis, along with impaired axon elongation. These findings indicate that RAC3-N92K’s activated state significantly disrupts cortical development, expanding the genetic and pathophysiological spectrum of NEDBAF.

RAC small GTPases, RAC1-3, are members of the RHO subfamily. They regulate cell adhesion, morphology, migration, and cell cycle progression by controlling intracellular signaling pathways and cytoskeletal dynamics (1). Resembling other small GTPases, RACs alternate between active (GTP-bound) and inactive (GDP-bound) states through conformational changes, primarily in the switch I and II regions, which serve as a platform for GTP-dependent effector recognition (2). The temporal and spatial regulation of the GTP/GDP-bound state is controlled by guanine nucleotide exchange factors (GEFs), which facilitate GDP/GTP exchange to shift the GTPase conformation toward the active state, and by GTPase-activating proteins (GAPs), which enhance intrinsic GTP-hydrolysis activity to shift the conformation toward the inactive state (3). Among the three members of the RAC subfamily, RAC1 and RAC3 are expressed in the central nervous system (CNS). RAC1 is expressed ubiquitously, whereas RAC3 expression is specific to the CNS (4, 5, 6). RAC3 plays a pivotal role in regulating neuronal migration and the development of axons, dendrites, and synapses (7, 8, 9, 10, 11).

While a variety of genes encoding molecules involved in RAC signaling pathways have been implicated in neurodevelopmental disorders (NDDs) (12, 13), recent studies have revealed that *de novo* deleterious variants in the human *RAC1* (MIM ∗ 602048) and *RAC3* (MIM ∗ 602050) genes are directly implicated as the cause of neurodevelopmental phenotypes (14, 15, 16, 17, 18, 19, 20, 21, 22). These findings confirm the critical role of RAC signaling in brain development and its contribution to the pathogenesis of NDDs (13). Notably, pathogenic *RAC1* variants have been associated with abnormalities in brain size, including microcephaly and macrocephaly (14, 15, 16, 17). In contrast, *RAC3* variants have been demonstrated to impede neuronal migration and result in malformations of cerebral cortex, such as polymicrogyria and heterotopia (18, 19, 20, 21, 22). The distinct neurodevelopmental phenotypes caused by gene abnormalities of *RAC1* or *RAC3*, despite their 92% amino acid sequence identity and shared downstream effectors, indicate that they play distinctive roles in brain development.

So far, 13 *de novo* missense variants in *RAC3* have been reported in patients with heterogeneous neurodevelopmental phenotypes featuring dysmorphism, developmental delay, cognitive dysfunction, seizures, and extensive structural brain abnormalities (18, 19, 20, 21, 22). These clinical manifestations are collectively referred to as Neurodevelopmental disorder with structural brain anomalies and dysmorphic facies (NEDBAF, MIM #618577). It is noteworthy that all pathogenic *RAC3* variants reported thus far are described as activated versions (18, 19, 20, 22), in contrast to pathogenic *RAC1* variants, which display a variety of activation states including dominant negative, constitutively active, and context-dependent versions (14, 15, 16). All disease-causing *RAC3* variants thus far reported are located in the highly conserved functional regions including P-loop, switch I/II, and G boxes, which lead to impairment of the intrinsic GTPase activity of RAC3, supporting their pathophysiological relevance to NEDBAF (18, 19, 20, 21, 22). However, a novel *de novo* variant in *RAC3* involving a residue localized outside the abovementioned functional regions has been very recently associated with a complex spectrum of brain malformations in a prenatal case, the (NM_005052.3): c.276T>A, p.(N92K) variant (23). This finding led us to consider the possibility that this variant affects mechanisms other than intrinsic GTPase activity.

Here, we provide the first experimental evidence demonstrating the pathogenicity of the p.N92K variant, a mutation uniquely located outside the known functional domains of RAC3. In this study, we investigated the pathophysiological significance of the p.N92K variant by elucidating the molecular and cellular mechanisms involved in the RAC3-related disorder. Our findings not only establish the disease-causing nature of this variant but also shed light on its underlying mechanisms. *In vitro* analyses demonstrated that this variant is biochemically and biologically activated. Structural analyses revealed that the variant residue is exposed on the outside, and this variant could impair the interaction with GAP and shift the conformational population of RAC3 toward the activated state compared to the wild type. On the other hand, this N92K variant did not impact the interaction with a GEF and the typical RAC3 effectors, PAK1 (p21-activated kinase 1) and MLK2 (Mixed lineage kinase 2; Mitogen-activated protein kinase kinase kinase 10, MAPKKK10). Subsequent *in vivo* studies showed that the resulting RAC3 dysfunction caused defects in cortical neuron migration and axon elongation. Taken together, we showed that the p.N92K variant disrupts regulation of the RAC3 activity and causes significant defects in corticogenesis, leading to the malformations of cortical development. Our findings suggest that *RAC3* variants localized outside the functional regions including P-loop, switch I/II, and G boxes may still be able to affect protein activity and cause human disease.

## Results

### Biological and biochemical properties of the p.N92K variant in *RAC3*

**Figure 1:**
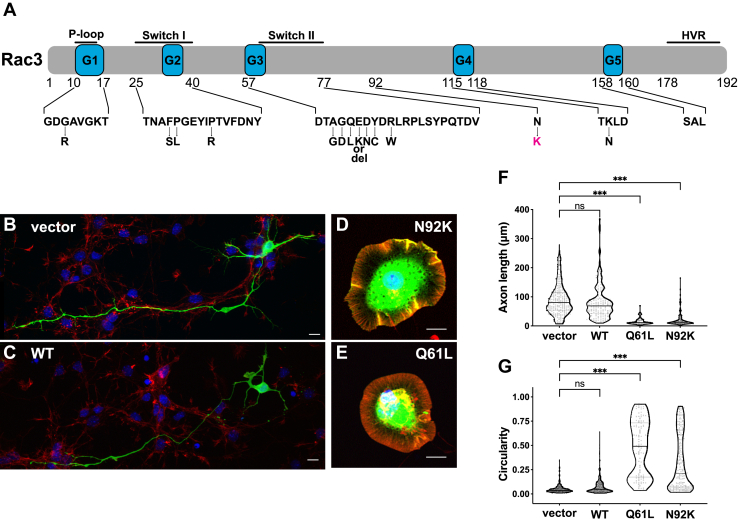
**Effects of the *RAC3* p.N92K variant on neuronal morphology *in vitro***. *A*, schematic representation of RAC3 structure showing the position of the variant identified in this study (p.N92K) in *magenta*, and previously reported variants (p.G12R, p.F28S, p.P29L, p.P34R, p.A59G, p.G60D, p.Q61L, p.E62K, p.E62del, p.D63N, p.Y64C, p.R66W, and p.K116N) in *black*. Note that the N92 residue is located outside of any known functional regions of the protein. HVR, hypervariable region. *B–E*, hippocampal neurons dissociated at E16 were co-electroporated with pCAG-EGFP (0.1 μg) and either pCAG-Myc (vector) (*B*), -Myc-RAC3 (*C*), -RAC3-N92K (*D*), or -RAC3-Q61L (*E*) (0.3 μg each). Cells were fixed at 3 days *in vitro* and co-stained with anti-GFP (*green*), rhodamine-phalloidin (*red*), and DAPI (*blue*). Representative images were shown. Scale bars, 10 μm. *F* and *G*, quantification of (*B*–*E*). *F*, axon length of neurons expressing pCAG-Myc (vector), -Myc-RAC3, -RAC3-N92K, or -RAC3-Q61L (≧ 62 cells each) was quantified. Statistical significance between vector and each variant was determined with Dunnett's test. vector *versus* WT, *p* = 0.43; vector *versus* Q61L, *p* < 0.001; vector *versus* N92K, *p* < 0.001. *G*, the morphological descriptor (circularity) of GFP-positive neurons (≧ 80 cells each) is shown in violin and dot plots. Statistical significance between vector and each variant was determined with Dunnett's test. vector vs. WT, *p* = 0.17; vector *versus* Q61L, *p* < 0.001; vector *versus* N92K, *p* < 0.001. ns: not significant, ∗∗∗*p* < 0.001.

All pathogenic *RAC3* variants identified to date are located in the functional regions including P-loop, switch I/II, and G boxes, and function as constitutively active versions *in vitro* (Fig. 1*A*) (18, 19, 20, 21, 22). To ascertain the characteristics of the p.N92K variant, we initially evaluated the activation status of RAC3-N92K in primary cultured hippocampal neurons. While wild-type RAC3 had minimal impact on neuronal differentiation (Fig. 1, *B*, *C*, *F*, and *G*), neurons expressing RAC3-N92K exhibited compromised differentiation, resulting in a round cellular shape with typical lamellipodia (Fig. 1, *D*, *F*, and *G*). This phenotype was similar to that caused by RAC3-Q61L, a typical constitutively active variant (Fig. 1, *E*–*G*). These findings indicate that RAC3-N92K represents a biologically activated version.

**Figure 2:**
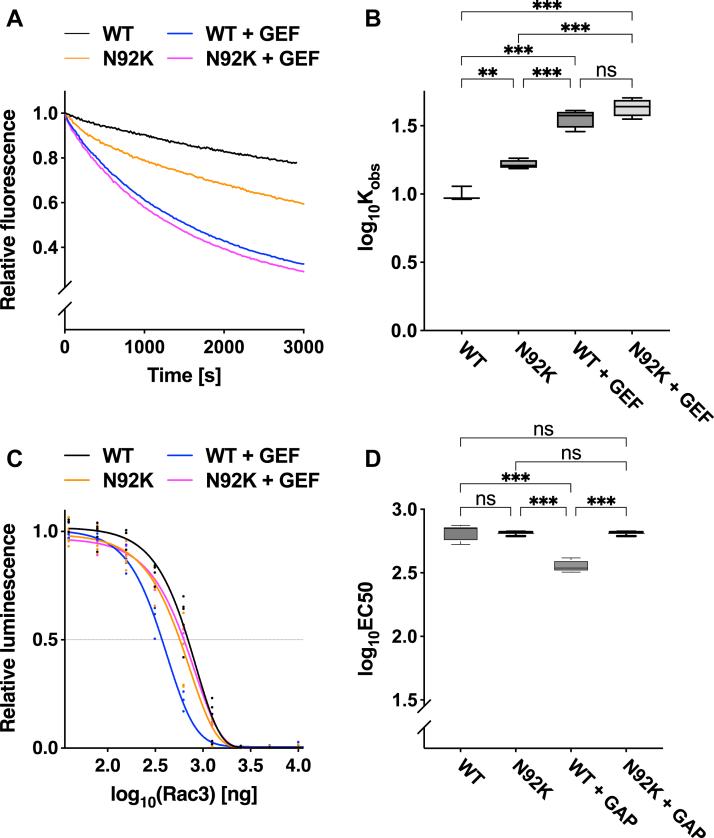
**Effects of the *RAC3* p.N92K variant on biochemical activation status**. *A* and *B*, measurement of GDP/GTP-exchange activity. *A*, recombinant His-tag-fused RAC3 (WT), RAC3-N92K, or RAC3-Q61L was preloaded with fluorescent mant-GDP and incubated with non-hydrolysable GTP analog. *B*, the ^mant^GDP-dissociation rates of each condition were calculated as observed rate constants (K_obs_ [×10^−5^ s^−1^]) from the results in (*A*). Number of replicates, N ≥ 4. Statistical significance between each condition was determined using Tukey's test. WT *versus* N92K, *p* = 0.002; WT *versus* WT + GEF (Trio-D1), *p* < 0.001; WT *versus* N92K + GEF, *p* < 0.001; N92K *versus* WT + GEF, *p* < 0.001; N92K *versus* N92K + GEF, *p* < 0.001; WT + GEF *versus* N92K + GEF, *p* = 0.26. *C* and *D*, measurement of GTP-hydrolysis activity. *C*, the intrinsic activity was analyzed by directly measuring changes in GTP concentration using the GTPase-Glo assay kit. *D*, the EC50 (half maximal effective concentration) was estimated from the sigmoidal fitting curve in (*C*). Number of replicates, N ≥ 4. Statistical significance between each condition was determined using Tukey's test. WT *versus* N92K, *p* > 0.99; WT *versus* WT + GAP (CHN1), *p* < 0.001; WT *versus* N92K + GAP, *p* > 0.99; N92K *versus* WT + GAP, *p* < 0.001; N92K *versus* N92K + GAP, *p* > 0.99; WT + GAP *versus* N92K + GAP, *p* < 0.001. ns: not significant, ∗∗*p* < 0.002, ∗∗∗*p* < 0.001.

Subsequently, we measured its GTP/GDP exchange and GTP-hydrolysis activities to examine the biochemical properties of RAC3-N92K. The exchange reaction of RAC3-N92K was higher than that of the wild type but showed significant enhancement in the presence of a GEF, reaching levels comparable to the wild type (Fig. 2, *A* and *B*). In contrast, this variant on its own showed no enhancement in GTP hydrolysis activity compared to the wild type (Fig. 2, *C* and *D*). Notably, this variant was resistant to a GAP when compared to the wild type (Fig. 2, *C* and *D*). Considering the observed morphology of hippocampal neurons (Fig. 1), we conclude that RAC3-N92K is autonomously activated and tends to remain in an active GTP-bound state.

### Structural considerations of the p.N92K variant in *RAC3*

**Figure 3:**
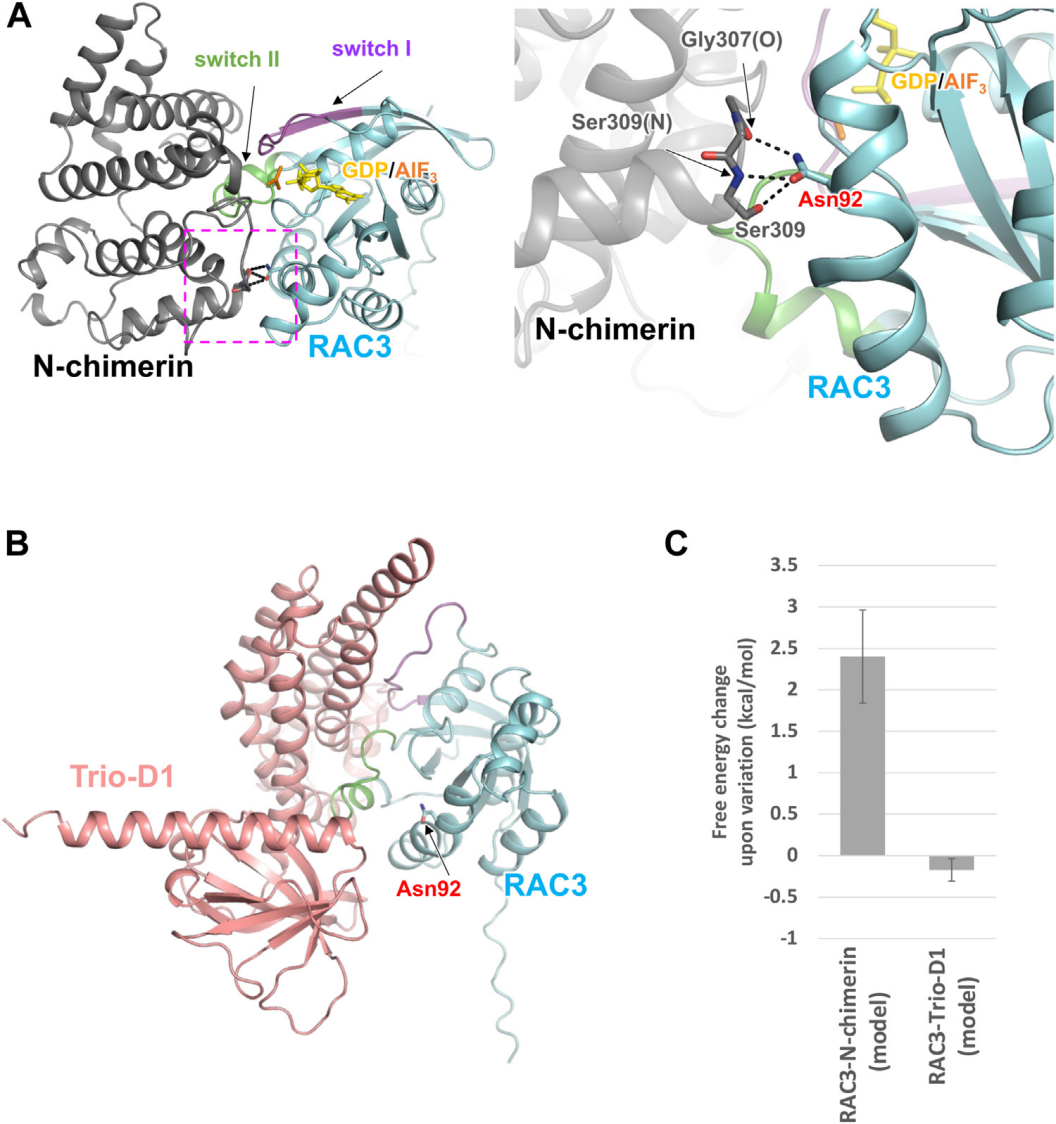
**Structural overview of RAC3 and its p.N92K variation position in the GAP-associated states**. *A*, the AlphaFold2 prediction model of the RAC3 (residues 1–192)-N-chimerin (residues 258–459) complex. The overall structure of the complex is shown on the *left*. A close-up view of the area enclosed by the magenta dashed line is shown on the *right*. Side chains of Asn92 of RAC3 (*cyan*) and Ser309 of N-chimerin (*gray*), and the backbone in the Gly307 to Ser309 region are shown as *stick models*. Labels (O) and (N) indicate the backbone oxygen and nitrogen atoms, respectively. A putative transition state mimic analog of the GTPase reaction, GDP-aluminum fluoride (GDP-AlF_3_), from the crystal structure of CDC42-GDP-AlF_3_-CDC42GAP complex is shown as *yellow* (GDP) and *orange* (AlF_3_) *sticks*. The switch I (residues 30–40) and switch II (residues 59–70) regions of RAC3 are shown in *magenta* and *green*, respectively. *Dotted black lines* indicate hydrogen bonds. *B*, alphaFold2-predicted model of the RAC3 (residues 1–192)-Trio-D1 (residues 1282–1606) complex. RAC3 is colored as in (*A*), while Trio-D1 is shown in salmon pink. *C*, free energy change resulting from the p.N92K variation. Free energy changes were calculated using the FoldX software, based on both the AlphaFold2-predicted models of the RAC3-N-chimerin and RAC3-Trio-D1 complexes.

Although RAC3-N92K is a constitutively active variant, the variation site is located outside the functional regions including P-loop, switch I/II, and G boxes. This suggests that the Asn92 residue may also play a critical role in formation of the higher-order structure including regulatory molecules such a GAP, which could be essential for the functional regulation of RAC3. To investigate this hypothesis, we constructed a structural model of RAC3 with N-chimerin (a GAP for RAC utilized in this functional analysis), which is not available in the Protein Data Bank (PDB), using the AlphaFold2 software (Fig. 3*A*). The reliability of the AlphaFold2 prediction model was shown to be high throughout the complex structure based on the predicted local distance difference test (pLDDT) score, which represents the confidence level of the structural prediction (Fig. S2*A*). RAC3 and N-chimerin are proteins homologous to CDC42 and CDC42GAP, respectively, according to amino acid sequence comparisons (Fig. S3). The Asn92 of RAC3 was completely conserved among the homologs (Fig. S3*A*). The root-mean-square deviation (RMSD) between the complex model structure comprising the RAC3 (amino acids 1–177) and N-chimerin (amino acids 266–459) regions and the corresponding crystal structure of the CDC42-GDP-AlF_3_-CDC42GAP complex (PDB: 1GRN) (Fig. S2*B*) (24), which adopts the transition state of the GTP hydrolysis reaction, is 1.97 Å. This indicates that the overall structure, including the Asn92 position of the RAC3-N-chimerin complex model, spatially well overlaps with the crystal structure of the CDC42-GDP-AlF_3_-CDC42GAP complex (Fig. S2*C*).

In the RAC3-N-chimerin complex model, the Asn92 side chain of RAC3 forms intermolecular hydrogen bonds with the side chain hydroxyl group and the backbone nitrogen of Ser309 as well as the backbone oxygen of Gly307 of N-chimerin. These backbone interactions result in the formation of a helix capping (N-capping) in N-chimerin (Fig. 3*A*, *right*). Therefore, the p.N92K variant would likely destabilize the interaction between RAC3 and N-chimerin due to the loss of hydrogen bonds and introduction of steric hindrance by the large side chain of the replaced Lys92. Such destabilization may lead to RAC3 being constitutively activated and unable to suppress the activation of its downstream effectors.

The free energy change resulting from the N92K variation in the RAC3-N-chimerin complex was calculated using the FoldX software. The obtained free energy change was 2.40 ± 0.56 kcal/mol, suggesting moderate structural destabilization in the variant complex (Fig. 3*C*), which corroborates our structural evaluation. Similarly, based on the known crystal structure of the CDC42-GDP-AlF_3_-CDC42GAP complex, the free energy change caused by the N92K variation of CDC42 was 1.14 ± 0.65 kcal/mol (Fig. S2*D*), showing a comparable trend to the RAC3-N-chimerin complex model. In support with these results, the intrinsic GTPase activity of the p.N92K variant of RAC3 is not enhanced by the GAP (Fig. 2, *C* and *D*).

To investigate the effect of the p.N92K variant on the structure of RAC3, we used AlphaFold3 to predict the structures of wild-type RAC3 and RAC3-N92K, and compared the two structures. The RMSD was 0.23 Å, indicating no significant structural changes (Fig. S6). Asn92 faces outward from an α-helix, suggesting that this variant has minimal impact on RAC3 folding and primarily affects its interaction with GAP.

There are no other residues near Asn92 that interact with a GAP. Moreover, a multiple sequence alignment and conservation analysis using the ConSurf program (25) showed that Asn92 is highly conserved, while the surrounding exposed residues are not (Fig. S7). This finding suggests that Asn92 is important for the interaction with N-chimerin GAP.

Next, to evaluate the impact of the RAC3 variation on GEF binding, we predicted a structural model of RAC3 complexed with Trio-D1 (a GEF for RAC utilized in this functional analysis), which is not available in the Protein Data Bank (PDB), using AlphaFold2 (Figs. 3*B* and S4*A*). The RMSD between the complex model of RAC3 (amino acids 1–177) and Trio-D1 (amino acids 1290–1594) and the crystal structure of the corresponding complex of RAC1 and Trio-D1 (PDB: 2NZ8) was 1.68 Å, indicating high structural similarity of these complexes, including the position of Asn92 (Fig. S4, *A*–*C*). In the RAC3-Trio-D1 complex model, Asn92 of RAC3 does not interact directly with Trio-D1, suggesting the p.N92K variant of RAC3 does not affect this complex formation. FoldX calculations showed a free energy change of −0.17 ± 0.14 kcal/mol upon the N92K variation (Fig. 3*C*), indicating no significant destabilization. Similarly, the RAC1 N92K variant of the RAC1-Trio-D1 complex exhibited a free energy change of −0.14 ± 0.08 kcal/mol (Fig. S4*D*), showing the same trend as for the RAC3 variant. Consistently, the GTP/GDP exchange reaction of the p.N92K variant of RAC3 by the GEF showed enhancement to a similar extent as that of the wild type (Fig. 2, *A* and *B*).

### Interaction of RAC3-N92K with downstream effectors

Cell biological and biochemical analyses (Figs. 1 and 2) indicate that the p.N92K variant of *RAC3* functions as a constitutively activated form. While NEDBAF is characterized by a recognizable pattern of neurodevelopmental features, affected individuals show variable manifestations within the disease spectrum (18, 19, 20, 21, 22). These patient-specific clinical symptoms are significantly influenced by the nature and location of disease-causing amino acid changes. Indeed, different RAC3 variants have demonstrated variant-type-dependent interaction profiles with a range of downstream effectors (20).

**Figure 4:**
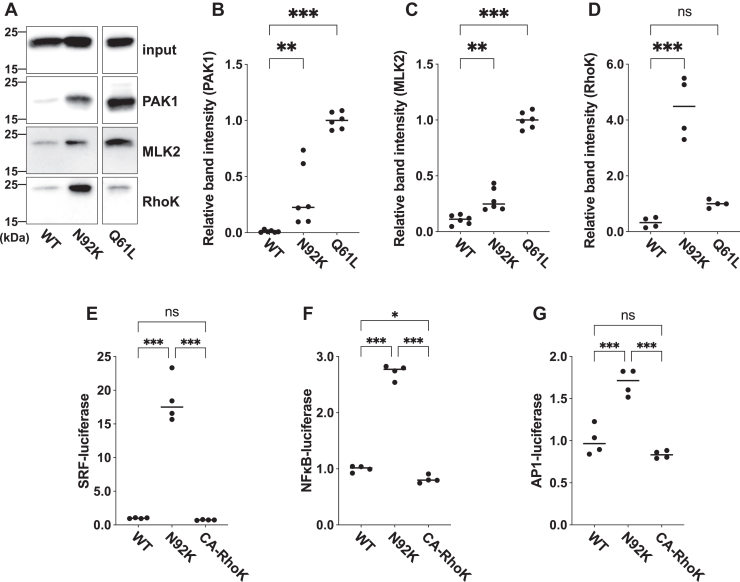
**Downstream signaling involved in the *RAC3* p.N92K variant**. *A–D*, interaction of RAC3-N92K with PAK1, MLK2, and Rho-kinase1 (RhoK). *A*, COS7 cells were transfected with pCAG-Myc-RAC3 (WT), RAC3-N92K, or -Q61L (0.3 μg each). A pull-down assay was conducted with respective GST-fused RBRs (5 μg each). 20 percent of the bound proteins was analyzed by western blotting (15% gel) using anti-Myc. Total cell lysates (3% of total volume) were also immunoblotted with anti-Myc for normalization (*input*). *B–D*, quantification of RAC3 bound to GST-RBR-PAK1 (*B*), -MLK2 (*C*), or -RhoK (*D*). The band intensity was measured by ImageJ software. The relative band intensity was expressed as a ratio relative to the value of RAC3-Q61L, which was set to 1.0. Data represent mean ± SEM (N ≧ 4). Statistical significance between WT and each variant was determined with Dunnett's test. *B*, WT *versus* N92K, *p* = 0.007; WT *versus* Q61L, *p* < 0.001. *C*, WT *versus* N92K, *p* = 0.002; WT *versus* Q61L, *p* < 0.001. *D*, WT *versus* N92K, *p* < 0.001; WT *versus* Q61L, *p* = 0.1. *E–G*, effects of the p.N92K variant and constitutively active Rho kinase 1 (CA-RhoK) on SRF- (*E*), NFκB- (*F*), and AP1-dependent (*G*) gene transcription. COS7 cells were co-transfected with each luciferase expression vector together with pCAG-Myc-RAC3 (WT), -RAC3-N92K, or CA-RhoK. Luciferase activity obtained with WT was set to 1.0, and relative activities are shown as Scatter plot. Data represent results from at least four independent experiments (N ≥ 4). Statistical significance between each condition was determined with Tukey's test. *E*, WT *versus* N92K, *p* < 0.001, WT *versus* CA-RhoK, *p* = 0.98, N92K *versus* CA-RhoK, *p* < 0.001. *F*, WT *versus* N92K, *p* < 0.001, WT *versus* CA-RhoK, *p* = 0.04, N92K *versus* CA-RhoK, *p* < 0.001. *G*, WT *versus* N92K, *p* < 0.001, WT *versus* CA-RhoK, *p* = 0.26, N92K *versus* CA-RhoK, *p* < 0.001. ns: not significant, ∗*p* < 0.033, ∗∗∗*p* < 0.001.

Resembling other RAC3 variants, the p.N92K variant is supposed to hyperactivate certain downstream effectors and dysregulates specific signaling pathways. To identify these pathways, we conducted pull-down assays to assess the interaction of RAC3-N92K with recombinant RBRs of downstream effectors PAK1, MLK2, and Rho-kinase 1. PAK1, a regulatory kinase involved in neuronal migration and implicated in NDDs (26, 27, 28, 29), and MLK2 (30, 31) exhibited moderate affinity with RAC3-N92K compared to RAC3-Q61L (Fig. 4, *A*–*C*). In contrast, Rho-kinase 1, a RHO-specific effector regulating actin cytoskeleton and cell polarity (32), demonstrated strong affinity towards RAC3-N92K, while RAC3-Q61L showed minimal affinity (Fig. 4, *A* and *D*).

We then analyzed the gene expression regulated by SRF, NFκB, and AP1, as RHO-family proteins are known to be involved in these signaling pathways (33, 34, 35). As shown in Figure 4, *E*–*G*, RAC3-N92K enhanced the transcription activity of these three genes compared to the wild type. The hyperactivation of PAK1, MLK2, and/or Rho-kinase 1 may influence the gene transcription pathways mediated by SRF, NFκB, and AP1. Since RAC3-N92K exhibits relatively high affinity for Rho-kinase 1, we tested the effect of Rho-kinase one on the transcriptional activity of SRF, NFκB, and AP1. However, a constitutively active version of Rho-kinase 1 (CA-RhoK) failed to activate these pathways, suggesting that unidentified effector(s) may be involved in regulating these transcriptional pathways (Fig. 4, *E*–*G*).

To investigate whether the p.N92K variant affects interactions with effectors, we referred the crystal structure of the RAC3-PAK1 complex (PDB: 2QME), which shows the interaction of the switch I region of RAC3 with the CRIB (Cdc42-and Rac-interactive binding) domain of PAK1 (Fig. S5*A*). Regarding the RAC3-MLK2 complex, we predicted it using AlphaFold3, which showed a binding mode similar to that observed in PAK1 (Fig. S5, *B* and *C*). In these complex structures, Asn92 of RAC3 does not directly involve binding to PAK1 or MLK2, suggesting the p.N92K variant of RAC3 does not impact these interactions. The free energy changes due to the N92K variation in the RAC3-PAK1 and the RAC3-MLK2 complexes are −0.37 ± 0.06 kcal/mol and 0.07 ± 0.11 kcal/mol, respectively, indicating no significant modulation for the interactions (Fig. S5*D*). Also, we attempted to predict the RAC3-RhoK complex using AlphaFold3. However, we could not obtain a reliable model. Further structural studies would be needed to clarify whether the N92K variant affects the complex formation of RAC3 with various effectors except for PAK1 and MLK2.

Taken together, structural analyses suggest that the p.N92K variant of RAC3 could destabilize the interaction with GAP, but not with GEF, resulting in shift of the conformational population of the RAC3 p.N92K variant toward the activated state compared to that of the wild-type RAC3.

### Effects of the p.N92K variant on neuronal migration during corticogenesis *in vivo*

**Figure 5:**
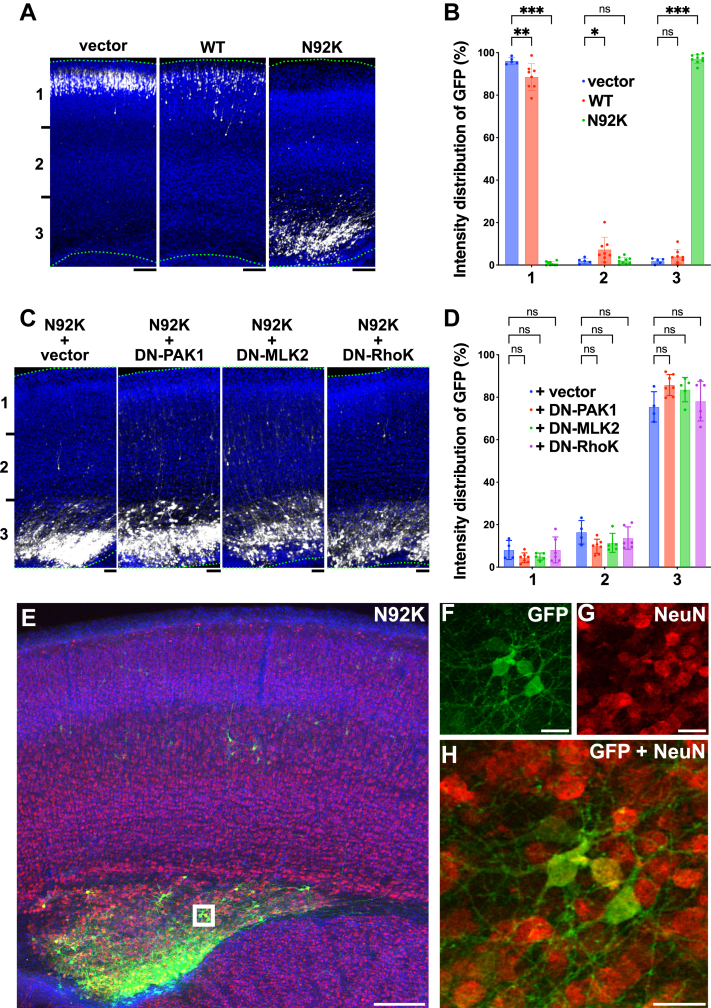
**Effects of the p.N92K variant on neuronal migration during corticogenesis *in vivo***. *A*, pCAG-EGFP (0.4 μg) was co-electroporated *in utero* with pCAG-Myc (vector), pCAG-Myc-RAC3 (WT), or -RAC3-N92K (0.1 μg each) into the VZ progenitor cells at E14.5. Coronal sections were prepared at P0 and double-stained with anti-GFP (*white*) and DAPI (*blue*). Scale bars, 100 μm. *B*, quantification of GFP-positive neuron distribution in distinct cortical regions (bins 1–3) for each condition in (*A*). Data represent results from at least five independent experiments (N ≥ 5). Statistical significance between vector and RAC3-expressing cells was determined using Dunnett's test for each bin. Bin 1: vector *versus* WT, *p* = 0.005; vector *versus* N92K, *p* < 0.001. Bin 2: vector *versus* WT, *p* = 0.04; vector *versus* N92K, *p* > 0.99. Bin 3: vector *versus* WT, *p* = 0.24; vector *versus* N92K, *p* < 0.001. *C*, rescue effects of dominant negative (DN) PAK1, MLK2, and Rho-kinase1 (RhoK) on migration defects. pCAG-Myc-RAC3-N92K (0.1 μg) was co-electroporated with pCAG-GFP (0.4 μg) together with control pCAG-Flag (vector), -PAK1-KA, -MLK2-KN, or -RhoK-RB/PH(TT) (RhoK) (1.0 μg each). Analysis was performed as in (*A*). Scale bars, 50 μm. *D*, quantification of (*C*). Distribution of GFP-positive neurons in cortical regions (bins 1–3) is shown as scatter plot with bar. Data represent results from at least four independent experiments (N ≥ 4). Statistical significance between vector and each rescue condition was determined using Dunnett's test for each bin. Bin 1: vector *versus* +DN-PAK1, *p* = 0.33; vector *versus* +DN-MLK2, *p* = 0.50; vector *versus* +DN-RhoK, *p* > 0.99. Bin 2: vector *versus* + DN-PAK1, *p* = 0.08; vector *versus* ++DN-MLK2, *p* = 0.26; vector *versus* + DN-RhoK, *p* = 0.66. Bin 3: vector *versus* + DN-PAK1, *p* = 0.07; vector *versus* ++DN-MLK2, *p* = 0.21; vector *versus* + DN-RhoK, *p* = 0.86. *E*–*H*, Long-term effects of RAC3-N92K. pCAG-Myc-RAC3-N92K was co-electroporated with pCAG-EGFP. Coronal sections were prepared at P7 and stained with anti-GFP (*Green*), DAPI (*Blue*), NeuN (*Red*). Boxed area in (*E*) was magnified to show GFP (*F*) and NeuN (*G*) signals. A merged image was presented in (*H*). Scale bars, 200 μm (*E*), 30 μm (*F*–*H*). *B* and *D*, statistical significance is denoted as follows: ns: not significant, ∗*p* < 0.033, ∗∗*p* < 0.002, ∗∗∗*p* < 0.001.

Resembling other *RAC3*-related disorder patients, polymicrogyria was an important finding in the prenatal NEDBAF case harboring the p.N92K variant (23), suggesting that this variant is linked to defective cortical neuron migration during corticogenesis. To test this hypothesis, we performed *in vivo* analyses to assess the impact of RAC3-N92K on the migration of newly born cortical excitatory neurons. We employed an *in utero* electroporation-mediated acute gene transfer method to co-transfect progenitor cells in the ventricular zone (VZ) of embryonic brains (E14) with pCAG-EGFP together with pCAG-Myc control vector, pCAG-Myc-RAC3, or pCAG-Myc-RAC3-N92K (0.1 μg each). The distribution of transfected cells and their progeny was analyzed at P0. Neurons transfected with pCAG-Myc migrated normally to the superficial layer (bin 1; layers II/III) of the cortical plate (CP), while expression of wild-type RAC3 resulted in a slight impairment of migration (Fig. 5, *A* and *B*). These results indicate that basal RAC3 activity had a minimal effect on neuronal migration. In contrast, the majority of cells expressing RAC3-N92K remained in the lower part of the CP (bin 3; VZ/subventricular zones [SVZ] and the intermediate zone [IZ]) (Fig. 5, *A* and *B*).

Given that RAC3-N92K interacts with PAK1, MLK2, and Rho-kinase 1, we sought to determine the potential contributions of these kinases to the migration anomalies induced by the variant. We performed *in utero* co-electroporation of pCAG-EGFP with pCAG-Myc-RAC3-N92K along with either pCAG-Flag-PAK1-KA, pCAG-Flag-MLK2-KN, or pCAG-Flag-RhoK-RB/PH(TT), which encode the dominant negative forms of PAK1, MLK2, and Rho-kinase 1, respectively. However, the positional defects of GFP-positive cells remained unchanged at P0 (Fig. 5, *C* and *D*), suggesting that these kinases may not be involved in the observed migration defects. These findings indicate that the signaling pathways dysregulated by RAC3-N92K differ from those affected by canonical *RAC3* variants specifically targeting the switch II region (*i.e.*, p.E62del, p.D63N, and p.Y64C), as the migration defects associated with these variants were effectively rectified by PAK1-KA (20). We then examined the long-term effects of RAC3-N92K expression and observed that abnormally positioned neurons formed large clusters at the VZ at P7 (Fig. 5*E*; 84.6%, n = 11/13 brains), similar to findings with other variants in the switch II region (20). Cells within these clusters frequently extended neurites and were NeuN-positive (Fig. 5, *F*–*H*), indicating differentiation at atypical locations. Collectively, these results suggest that RAC3-N92K impairs cortical neuron migration rather than merely delaying it, ultimately leading to heterotopia and/or polymicrogyria, commonly seen in patients with *RAC3*-related disorders.

### Effect of the p.N92K variant on axon extension during corticogenesis *in vivo*

**Figure 6:**
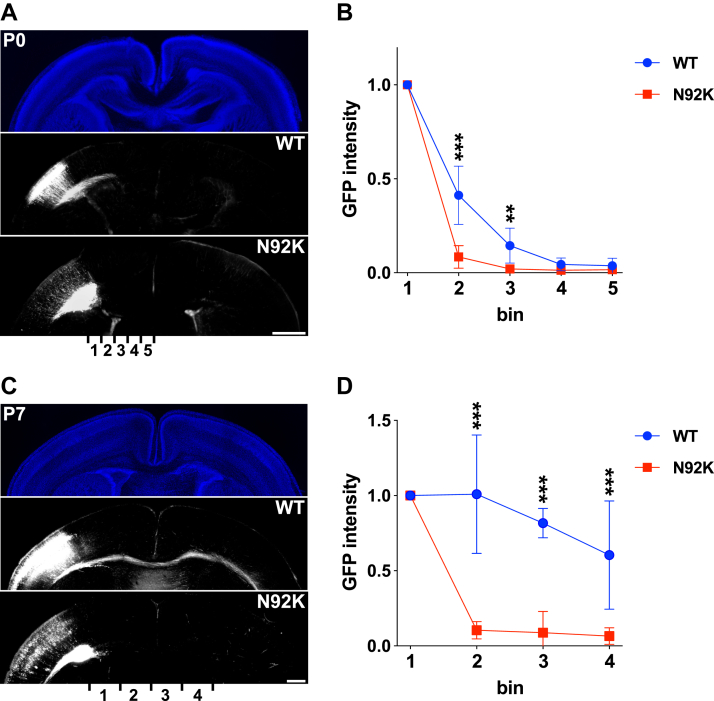
**Effects of the *RAC3* p.N92K variant on axon extension during corticogenesis *in vivo***. *A* and *C*, pCAG-GFP (0.4 μg) was co-electroporated with pCAG-Myc-RAC3 (WT) or -RAC3-N92K (0.1 μg each) into the VZ progenitor cells at E14.5. Coronal sections were prepared at P0 (*A*) or P7 (*C*), and stained with anti-GFP (*white*). DAPI staining (*blue*) is shown in *top panels*. Scale bars, 500 μm. *B* and *D*, quantification of GFP intensity in callosal axons at P0 (*B*) or P7 (*D*) in different regions (bins 1–five for P0 and bins 1–four for P7). Relative intensities were normalized with bin one set to 1.0. Data represent results from at least four independent experiments (N ≥ 4). Statistical significance was determined using two-way ANOVA [B: F (3, 34), *p* < 0.001, D: F (4, 49), *p* < 0.001] followed by Šidák's *post hoc* test. *B*, bin 2, *p* < 0.001; bin 3, *p* = 0.008; bin 4, *p* = 0.93; bin 5, *p* = 0.99. *D*, bin 2, *p* < 0.001; bin 3, *p* < 0.001; bin 4, *p* < 0.001. Statistical significance is denoted as follows: ∗∗*p* < 0.002, ∗∗∗*p* < 0.001.

All previously reported patients with NEDBAF exhibited callosal anomalies (18, 19, 20, 21, 22) with one exception (23). Consistently, our previous *in vivo* mouse analyses demonstrated impaired axon elongation in five pathogenic *RAC3* variants (p.F28S, p.Q61L, p.E62del, p.D63N, and p.Y64C) (20, 22). We here investigated the effect of the p.N92K variant on axon elongation in layer II/III pyramidal neurons during corticogenesis. We electroporated wild-type RAC3 and RAC3-N92K into the VZ progenitor cells at E14 and visualized the axon bundle of the corpus callosum at P0 and P7. In contrast to the control cells, which extended axon bundles towards the contralateral cortex, neurons expressing RAC3-N92K exhibited minimal axon elongation at P0 (Fig. 6, *A* and *B*). Further analysis at P7 revealed that axon elongation was not detected in neurons expressing RAC3-N92K, whereas control cells exhibited axon bundle extension into the contralateral cortex (Fig. 6, *C* and *D*). These findings suggest that RAC3-N92K inhibits, rather than merely delays, axon elongation in cortical neurons in our mouse model, although callosal abnormalities were not observed in the prenatal case with the p.N92K variant (23).

## Discussion

Deleterious *de novo* variants in the human *RAC3* gene cause NEDBAF, a complex neurodevelopmental syndrome with dysmorphism, delayed psychomotor development, cognitive dysfunction, seizures, and extensive malformations of cortical development (18, 19, 20, 21, 22, 23). All previously reported pathogenic *RAC3* variants are constitutively activated and are located in the functional regions including P-loop, switch I/II, and G boxes (20, 22). These regions are involved in the GTP/GDP-exchange and the intrinsic GTPase activities of RAC3, the interactions with modulatory molecules such as GAPs and GEFs that determine the ratio of the GTP/GDP-bound states, and the effectors that constitute the downstream RAC3 signaling. In contrast, although the N92 position is highly conserved among RHO family small GTPases, it is located outside the functional regions, including the P-loop, switch I/II, and G boxes. Therefore, this variant is considered noncanonical.

We investigated the impact of the p.N92K variant on the function and activity of RAC3. Overexpression of RAC3-N92K induced typical lamellipodia in primary hippocampal neurons, indicating that this variant is biologically activated (Fig. 1). Subsequent biochemical analyses indicated that RAC3-N92K impaired the enhancement of the intrinsic GTPase activity by a GAP (CHN1) due to lack of GAP interaction (Fig. 2), showing that RAC3-N92K is activated. Upon investigation of the interaction between RAC3-N92K and effector molecules PAK1, MLK2, and Rho-kinase1, the variant exhibited varying affinities towards each effector (Fig. 4, *A*–*D*). Notably, RAC3-N92K demonstrated a greater affinity toward an RHO-specific downstream effector, Rho-kinase 1, compared to the canonical RAC effectors, PAK1 and MLK2. To date, no *RAC3* variant has been reported to demonstrate this unique interaction pattern (20, 22).

Structural analyses suggest that the p.N92K variant destabilizes the interaction with GAP (CHN1), leading to constitutive activation of RAC3, but does not impair binding to GEF (Trio-D1) or several effectors, such as PAK1 and MLK2 (Figs. 3, S4, and S5). The N92K variation position is structurally exposed toward outside, therefore, this variation may alter the interface to be preferable for interacting with some effectors such as Rho-kinase 1, potentially contributing to its distinct functional impact.

Each *RAC3* variant results in the dysregulation of downstream effector systems and activation of gene expression machineries, including AP1, SRF, and NFκB, depending on the type of variant. This should lead to variant-specific pathophysiological and clinical phenotypes. RAC3-N92K was demonstrated to enhance all the tested AP1, SRF, and NFκB signaling pathways, thus resembling RAC3-G12R, -A59G, -Q61L, -E62del, -D63N, and -Y64C (20). While these similarities may account for the common cerebral malformations observed in the subject harboring the p.N92K variant and previously reported *RAC3*-related disorder patients, the molecular mechanisms underlying gene expression remain to be elucidated. Indeed, it is likely that the downstream effectors functioning under respective variants significantly differ. Further investigation will be important to dissect the pathophysiological significance of different *RAC3* variants on downstream signaling pathways at the molecular level.

Callosal abnormalities are common in patients with *RAC3*-related disorders (18, 19, 20, 21, 22), and the p.N92K variant also demonstrated impaired axon elongation in both *in vitro* and *in vivo* experiments (Figs. 1 and 6). Interestingly, however, the subject harboring the p.N92K variant represents the first documented case of NEDBAF without callosal abnormalities (23), which appears to contradict our experimental findings. This discrepancy might be attributable to the unique characteristics of the p.N92K variant or additional modulatory factors such as genetic background and compensatory mechanisms. It would be valuable to investigate whether the p.N92K variant is consistently associated with an absence of corpus callosum defects or if the reported case should be regarded as an exception. Although this investigation may be challenging due to the rarity of NEDBAF, reports of novel noncanonical *RAC3* variants could be essential to determine any possible association with a normal corpus callosum, in contrast to classic NEDBAF patients. Furthermore, future studies exploring compensatory mechanisms that may be activated in the presence of the p.N92K variant or other noncanonical variants could yield important insights into the variability of callosal phenotypes in RAC3-related disorders.

*In utero* electroporation-mediated acute expression of RAC3-N92K in cortical neurons resulted in migration defects, abnormal positioning, and neuronal clusters resembling subventricular heterotopia. Rescue experiments showed that the three effectors interacting with p.N92K did not seem to contribute to these migration abnormalities, despite PAK1’s known involvement in similar phenotypes associated with other variants, such as p.E62del, p.D63N, p.Y64C, and p.F28S (20, 22). Notably, p.N92K hyperactivates RAC3 both *in vitro* and *in vivo*. Identifying the downstream effectors responsible for these unique phenotypes will be essential to uncover the subtle molecular mechanisms underlying NEDBAF-associated brain development defects.

Our study demonstrates that the p.N92K variant in *RAC3* significantly disrupts key brain development processes, contributing to the cortical malformations typical of NEDBAF. Biochemical and biological analyses confirmed a gain-of-function effect, as p.N92K acts as a hyperactive form of RAC3 *in vitro*. Additionally, *in vivo* studies revealed the inhibition of cortical neuron migration and axon extension. These findings expand the genotype and pathophysiological spectrum of *RAC3* variants to include noncanonical changes outside the typical functional regions, such as the P-loop, switch I/II, and G boxes. Structural analysis indicated that the outwardly exposed p.N92K residue may lose GAP-mediated enhancement of the intrinsic GTPase activity of RAC3, resulting in impaired inactivation. Further studies are crucial to elucidate the downstream signaling pathways disrupted by noncanonical RAC3 variants, enhancing our understanding of the complex clinical manifestations of NEDBAF and paving the way for novel targeted therapies.

## Experimental procedures

### Plasmids

Human *RAC3* was obtained as described (22) and cloned into pCAG-Myc vector. Site-directed mutagenesis was carried out to generate RAC3-N92K and RAC3-Q61L, a constitutively activated version which is also pathogenic (19, 20), using KOD-Plus Mutagenesis kit (Toyobo Inc) and pCAG-Myc-RAC3 as the template. *RAC3* and its variants were also constructed into the pTriEx-4 vector (Merck). The cDNAs of PAK1-KA, a kinase-negative version featuring the single aa substitution p.K299A, and MLK2-KN, a kinase-negative variant lacking amino acids 139 to 183, were generous gifts from the late Prof. Alan Hall (Univ. College London). Rho-kinase 1 and RhoK-RB/PH(TT), a C-terminal fragment of Rho-kinase 1, that inhibits endogenous Rho-kinase 1, were gifted from Prof. Kozo Kaibuchi (Fujita Medical Univ) (36). Constitutively active Rho-kinase 1 (CA-RhoK), consisting of the kinase domain of Rho-kinase 1 (amino acids 6–553), was generated by PCR. These kinase mutants were subcloned into a pCAG-Flag vector. pGS21a vectors (GenScript) containing the RAC-binding region (RBR) of PAK1 (amino acids 67–150), MLK2 (amino acids 401–550), or Rho-kinase 1 (amino acids 67–150) were prepared as described previously (22, 36). The DH/PH domain (aa 1244–1969) of mouse Trio (Trio-D1, a RAC-GEF) and α1-chimerin (CHN1, a RAC-GAP) were obtained as described (17). For the purpose of gene transcription analysis, pGL4.74[hRluc/TK] (control reporter plasmid), pGL4.44[luc2P/AP1-RE/Hygro] (AP1-luciferase reporter plasmid), and pGL4.32[luc2P/NF-κB-RE/Hygro] (NFκB-luciferase reporter plasmid) were purchased from Promega (Madison, WI). The SRF-luciferase reporter plasmid was synthesized as described (22). All constructs were confirmed *via* DNA sequencing.

### Antibodies and histochemical reagents

Anti-GFP (Medical & Biological Laboratories, Nagoya, Japan, Cat# 598, RRID: AB_591819; and Nacalai Tesque, Cat# 04404-84, RRID: AB_10013361), anti-NeuN (Millipore, Temecula, CA, Cat# MAB377, RRID: AB_2298772), and anti-Myc (Medical & Biological Laboratories, Nagoya, Japan, Cat# M047-3, RRID: AB_591112) were used. For secondary antibodies, we employed Alexa Fluor 488 and 568 (Invitrogen). 4′,6-diamidino-2-phenylindole (DAPI; Nichirei Bioscience) and Rhodamine-phalloidin (Invitrogen) were used for staining DNA and F-actin, respectively.

### GTP/GDP-exchange and GTP-hydrolysis assays

His-tag-fused RAC3, RAC3-Q61L, RAC3-N92K, Trio-D1, and CHN1 proteins were prepared and purified according to the manufacturer’s instructions (QIAGEN) (37). To assess basal GTP/GDP-exchange activity, we measured the release of methylanthraniloyl (mant)-GDP (Sigma-Aldrich) as previously described (38). Intrinsic GTP-hydrolysis activity was measured using the GTPase-Glo Assay Kit (Promega) following the manufacturer’s instructions (39).

### Structural analyses

To evaluate the structural impact of the p.N92K variant on the interaction with N-chimerin as a GAP and Trio-D1 as a GEF, we used AlphaFold2 (40) to predict the models of the RAC3-N-chimerin and the RAC3-Trio-D1 complexes, respectively. This model prediction was facilitated through the use of ColabFold (41), an open source software that integrates the rapid homology search of MMseqs2 (42) with AlphaFold2 to accelerate predicting protein structures including complexes. For evaluation of the structural effects of the p.N92K variant on its interactions with effectors, we referred to the crystal structure of the RAC3-PAK1 complex (PDB: 2QME) and predicted the GTP-bound RAC3-MLK2 complex using AlphaFold 3 (43). The FoldX program (44, 45) was used to calculate the free energy change resulting from the RAC3 variation. ESPript 3.0 (46) was employed for amino acid sequence alignment. PyMOL (Schrödinger, LLC, http://www.pymol.org) was used for molecular comparison and molecular imaging.

### Cell culture and transfection

COS7 and primary hippocampal neurons derived from embryonic day (E) 16.5 mice were cultured according to the previous methods (47). Transient transfection into COS7 cells and neurons was carried out using polyethyleneimine “MAX” reagent (Polysciences Inc) and Neon transfection system (Invitrogen), respectively.

### Pull-down assay

Glutathione S-transferase (GST)-fused RBRs of PAK1, MLK2, and Rho-kinase1 were expressed in the *Escherichia coli* BL21 (DE3) strain and purified according to the manufacturer’s instructions. COS7 cells were transfected with pCAG-Myc-RAC3, -RAC3-N92K, or -Q61L (1.0 μg/60 mm-dish). After 24 h, cells were lysed with the pull-down buffer (50 mM Tris-HCl, pH7.5, 150 mM NaCl, 5 mM MgCl_2_, 0.1% SDS, 1% Nonidet P-40, and 0.5% deoxycholate). After insoluble materials were removed by centrifugation, the supernatant was incubated for 30 min at 4 °C with Glutathione-Sepharose 4B beads (GE Healthcare Life Sciences) conjugated to GST-RBR of PAK1, MLK2, or Rho-kinase1. The bound proteins were then analyzed using western blotting and the LAS-4000 luminescence image analyzer (GE Healthcare Life Sciences). Uncropped blotting data were shown in Fig. S1.

### Assay of SRF-, NFκB-, or AP1-mediated gene transcription

The assays were performed according to the established protocols (48). Briefly, COS7 cells were seeded on 24-well plates and transfected with pCAG-Myc-RAC3, pCAG-RAC3-N92K, or pCAG-CA-RhoK (0.05 μg/well) together with the control, SRF-, NFκB-, or AP1-luciferase reporter plasmid (0.2 μg/well) in various combinations. After 24 h, the cells were washed once with phosphate-buffered saline and harvested with the lysis buffer. A dual-luciferase reporter assay system (Promega) was used to determine luciferase activity, which was then normalized to the control vector.

### *In utero* electroporation

*In utero* electroporation was conducted as described (49, 50, 51). Briefly, pregnant ICR mice (Japan SLC, Shizuoka, Japan) were anesthetized deeply at embryonic day (E)14 with a combination of medetomidine (0.75 mg/kg), midazolam (4 mg/kg), and butorphanol (5 mg/kg) (52). Then, 1 μl of solution containing indicated amounts of plasmids was injected into the lateral ventricle of the embryos using a glass micropipette (GD-1; Narishige, Tokyo, Japan). After the embryo in the uterus was placed between the tweezers-type disk electrode (CUY650-5; NEPA Gene), electronic pulses (50 ms of 35 V) were applied 5 times at 450 ms intervals using an NEPA21 electroporator (NEPA Gene) to introduce plasmids into the somatosensory area of the parietal lobe. Brains were then fixed, sectioned and analyzed at the indicated time points. To quantify the distribution of GFP-positive cells, coronal sections were divided into three bins and fluorescence intensities at bins 1, 2, and 3 were quantified (at least three sections per brain). All experimental procedures were performed during the day. Animals were neither excluded nor died during experiments.

### Quantitative analysis of axon growth

To estimate axon growth, we measured GFP signal intensity of the callosal axons at P0 or P7 in specific regions (bins 1–5 for P0 analyses, and bins 1–4 for P7) using imageJ software. We normalized the relative intensities of the bins with bin 1 as 1.0 and compared them using R software.

### Immunofluorescence

The analysis was performed essentially as described (22). Images of cultured cells were captured using either a BZ-9000 (Keyence, Osaka, Japan) or an LSM-880 confocal laser microscope (Carl Zeiss). For cortical slice staining, brains were embedded in 3% agarose, sectioned into slices (100 μm thick) using a vibratome, and photographed with LSM-880. Acquired images were analyzed using ImageJ to determine cell morphological descriptors and fluorescence intensity.

### Statistical analysis

For all cell imaging experiments, cell selection and traces were assessed in a blinded manner using ImageJ software. Statistical significance for multiple comparisons was determined by Dunnett's or Tukey's test. Comparisons between two groups were performed with Welch's *t* test. *p* < 0.033 was considered statistically significant. Statistical analyses were performed using Prism 9 (GraphPad Software).

### Ethics approval

We adhered to the fundamental guidelines for proper conduct of animal experiments and related activity in academic research institutions under the jurisdiction of the Ministry of Education, Culture, Sports, Science, and Technology (Japan). All protocols for animal handling and treatment were reviewed and approved by the animal care and use committee of Institute for Developmental Research, Aichi Developmental Disability Center (approval number: 2019-013).

## Institutional review board statement

All protocols for animal handling and treatment were reviewed and approved by the animal care and use committee of Institute for Developmental Research, Aichi Developmental Disability Center (approval number: 2019-013).

## Data availability

The data that support the findings of this study are available from the corresponding authors, upon reasonable request.

## Supporting information

This article contains supporting information.

## Conflicts of interests

The authors declare that they have no conflicts of interest with the contents of this article.
