# Supplementary material for: A p.N92K variant of the GTPase RAC3 disrupts cortical neuron migration and axon elongation
Source: J Biol Chem. 2025 Feb 25;301(4):108346. doi: 10.1016/j.jbc.2025.108346 (PMC11968283; doi:10.1016/j.jbc.2025.108346)
Supplement: Supplementary 1 [file mmc1.pdf]

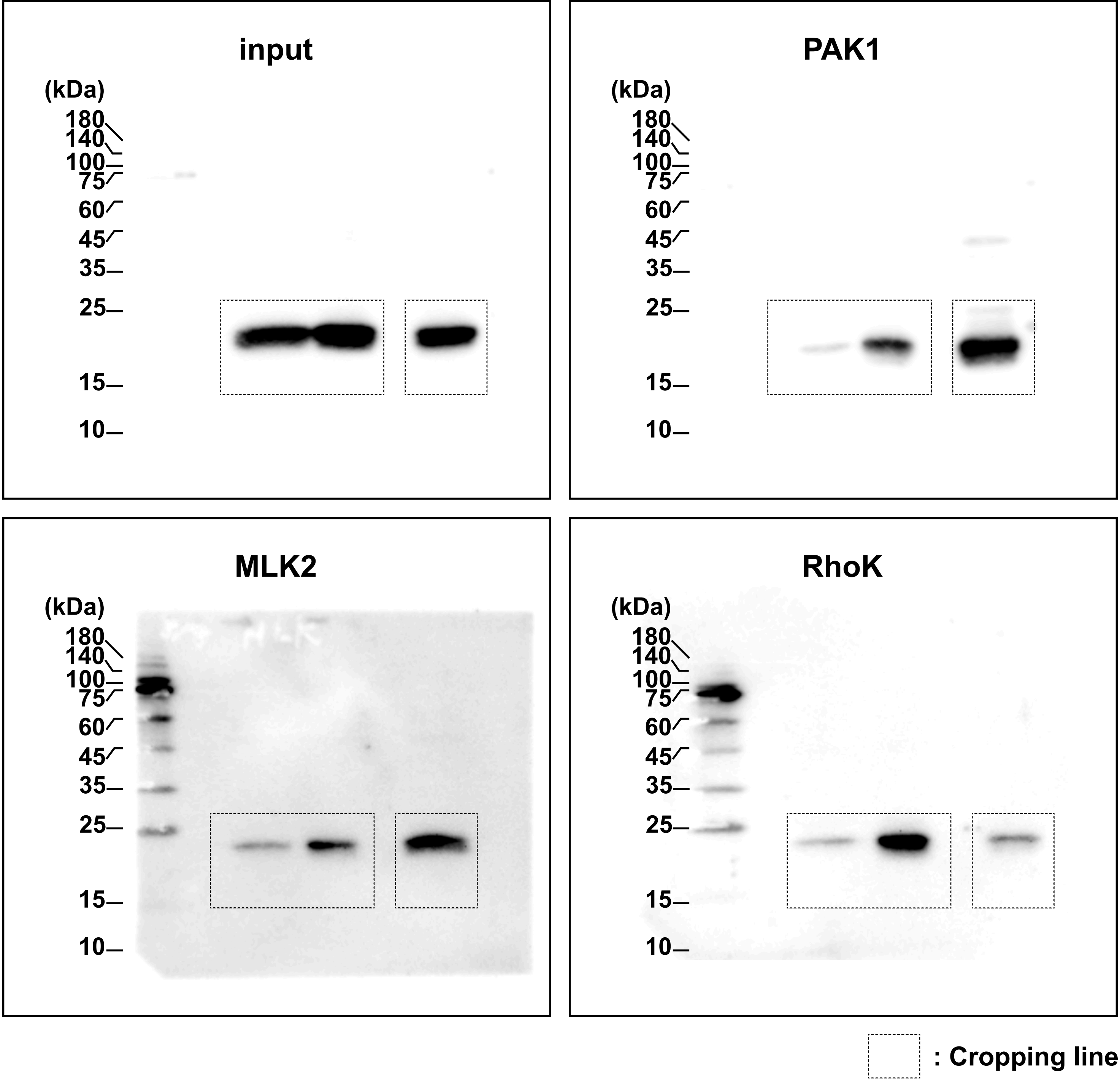

Supplementary Fig. 1. Uncropped blotting data of the pull-down assay
The data in Fig. 1A were prepared by cropping of the original blots in this figure.
