# Supplementary material for: A p.N92K variant of the GTPase RAC3 disrupts cortical neuron migration and axon elongation
Source: J Biol Chem. 2025 Feb 25;301(4):108346. doi: 10.1016/j.jbc.2025.108346 (PMC11968283; doi:10.1016/j.jbc.2025.108346)
Supplement: Supplementary 2 [file mmc2.pdf]

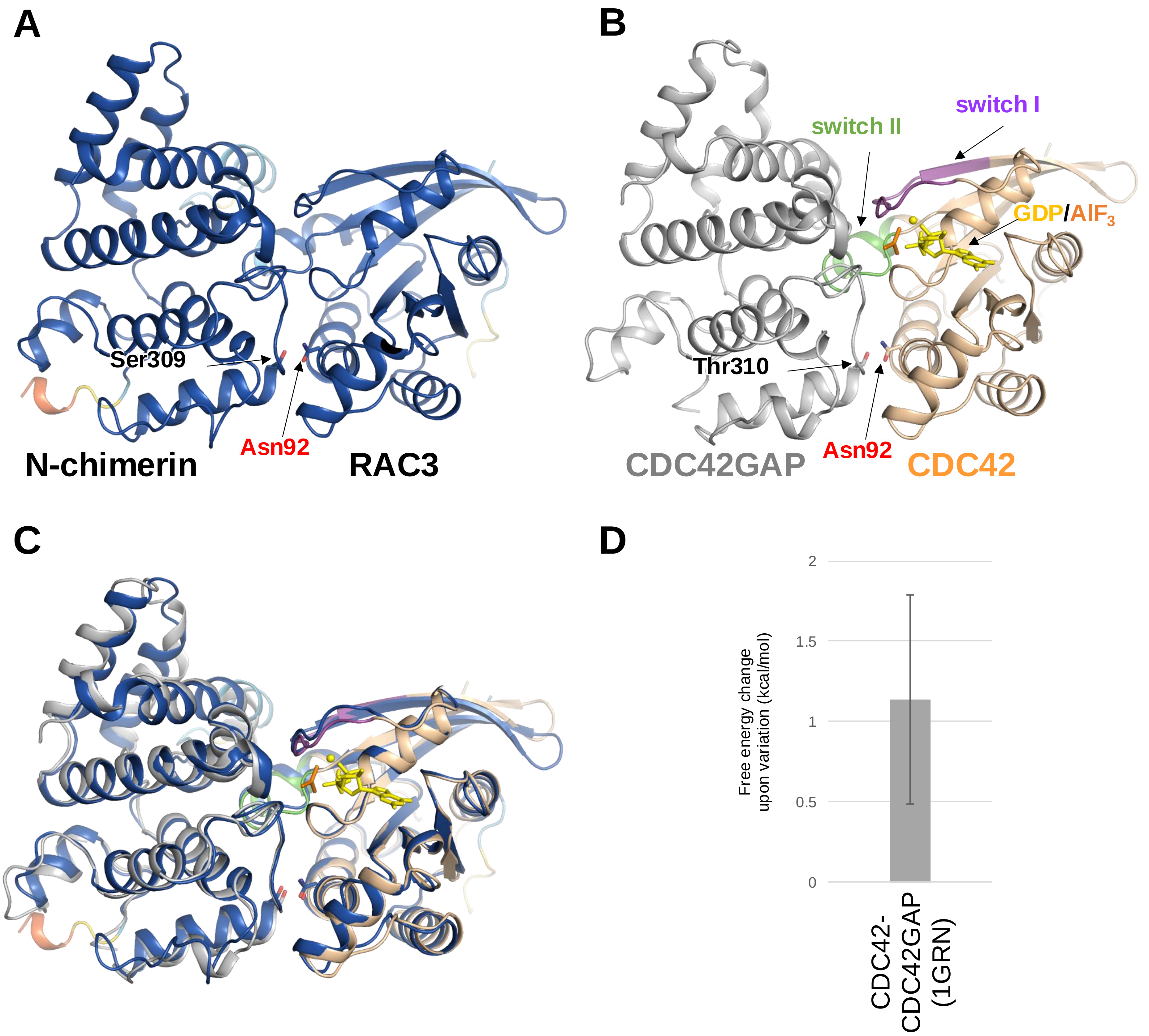

Supplementary Fig. 2. Structural overviews of the AlphaFold2-predicted model of the RAC3-N-chimerin complex and the crystal structure of the CDC42-GDP-AlF3-CDC42GAP complex (PDB: 1GRN)

(A) AlphaFold2-predicted model of the RAC3 (residues 1-192)-N-chimerin (residues 258-459) complex. The prediction reliability is indicated by the predicted local distance difference test (pLDDT) values, ranging from 0 to 100. Backbone structures are colored according to the confidence score (pLDDT): very high (90-100) in blue, high (70-90) in cyan, low (50-70) in yellow, and very low (0-50) in orange. Asn 92 of RAC3 and Ser309 of N-chimerin with the highest confidence score (blue) are shown by the stick model. (B) Crystal Structure of the CDC42 (residues 1-192)-GDP-AlF3-CDC42GAP (residues 237-433) complex (PDB: 1GRN). Asn92 of CDC42 (wheat) and Thr310 of CDC42GAP (light gray) are shown by the stick model. A putative transition state mimic analog of the GTPase reaction, GDP-aluminum fluoride (GDP-AlF_3), from the crystal structure of CDC42-GDP-AlF_3-CDC42GAP complex is shown as yellow (GDP) and orange (AlF_3) sticks. The switch I (residues 30-40) and switch II (residues 59-70) regions of CDC42 are shown in magenta and green, respectively. (C) Superimposed structures of (A) and (B). (D) Free energy change upon the p.N92K variation of CDC42 in the crystal structure of the CDC42-GDP-AlF_3-CDC42GAP complex.
