# Supplementary material for: A p.N92K variant of the GTPase RAC3 disrupts cortical neuron migration and axon elongation
Source: J Biol Chem. 2025 Feb 25;301(4):108346. doi: 10.1016/j.jbc.2025.108346 (PMC11968283; doi:10.1016/j.jbc.2025.108346)
Supplement: Supplementary 3 [file mmc3.pdf]

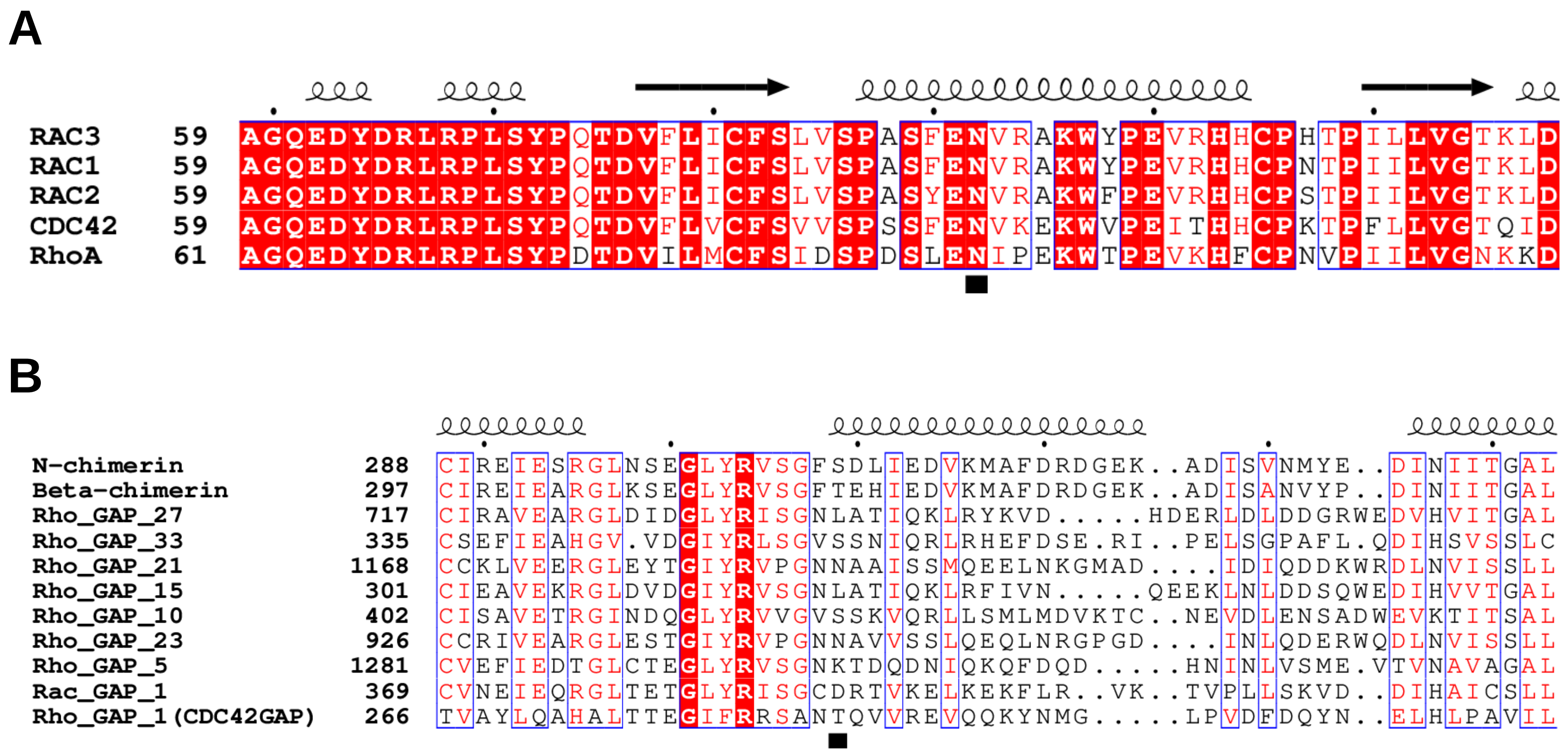


**Supplementary Fig. 3. Sequence alignments of GTPases and GAPs**
(A) Sequence alignment of RAC3 (residues 59-118) and selected homologs. The black closed square below the sequences indicates the position of N92 of RAC3. The indications of the β-strands (arrows) and α-helices (coils) adopted by CDC42 in the CDC42-GDP-$AlF_3$-CDC42GAP complex (PDB 1GRN) are shown above the sequence. (B) Sequence alignment of N-chimerin (residues 288-343) and selected homologs. The black closed square below the sequences indicates the position of S309 in N-chimerin, which interacts with N92 of RAC3.
