# Supplementary material for: A p.N92K variant of the GTPase RAC3 disrupts cortical neuron migration and axon elongation
Source: J Biol Chem. 2025 Feb 25;301(4):108346. doi: 10.1016/j.jbc.2025.108346 (PMC11968283; doi:10.1016/j.jbc.2025.108346)
Supplement: Supplementary 4 [file mmc4.pdf]

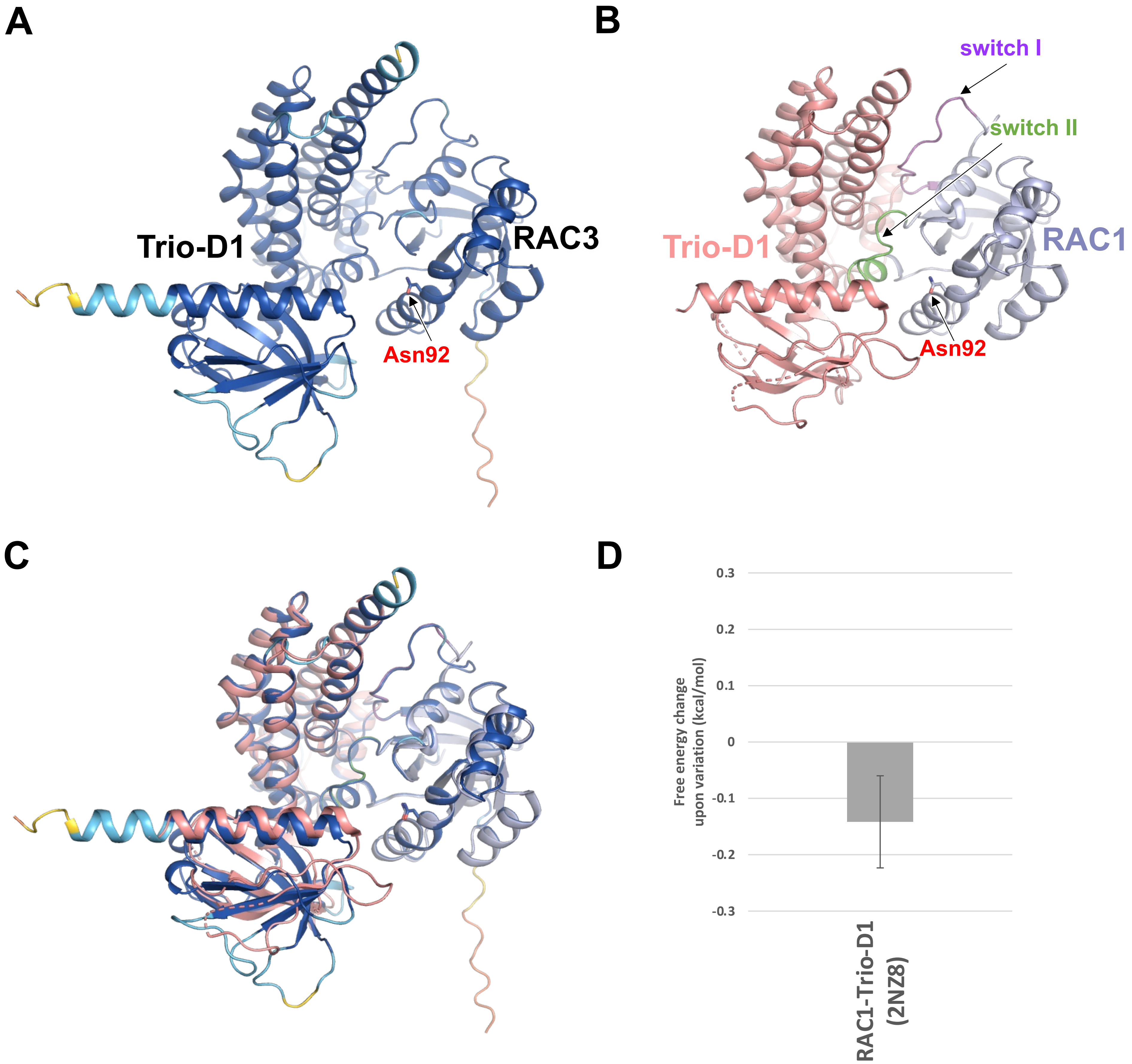

Supplementary Fig. 4. Structural overviews of the AlphaFold2-predicted model of the RAC3-Trio-D1 complex and the crystal structure of the RAC1-Trio-D1 complex (PDB: 2NZ8).

(A) AlphaFold2-predicted model of the RAC3 (residues 1-192)-Trio-D1 (residues 1282-1606) complex. Backbone structures are colored according to the confidence score (pLDDT) (see the legend of Supplementary Fig. 1). Asn 92 of RAC3 with the highest confidence score (blue) is shown by the stick model. (B) Crystal structure of the RAC1 (residues 1-177)-Trio-D1 (residues 1231-1535) complex (PDB: 2NZ8). RAC1 is shown in pale blue with Asn 92 depicted as the stick model, while Trio-D1 is shown in salmon pink. The switch I (residues 30-40) and switch II (residues 59-70) regions of RAC1 are shown in magenta and green, respectively. (C) Superimposed structures of (A) and (B). (D) Free energy changes upon the p. N92K variation of RAC3 and RAC1 in the model of the RAC3-Trio-D1 complex and the crystal structure of the RAC1-Trio-D1 complex, respectively.
