# Supplementary material for: A p.N92K variant of the GTPase RAC3 disrupts cortical neuron migration and axon elongation
Source: J Biol Chem. 2025 Feb 25;301(4):108346. doi: 10.1016/j.jbc.2025.108346 (PMC11968283; doi:10.1016/j.jbc.2025.108346)
Supplement: Supplementary 5 [file mmc5.pdf]

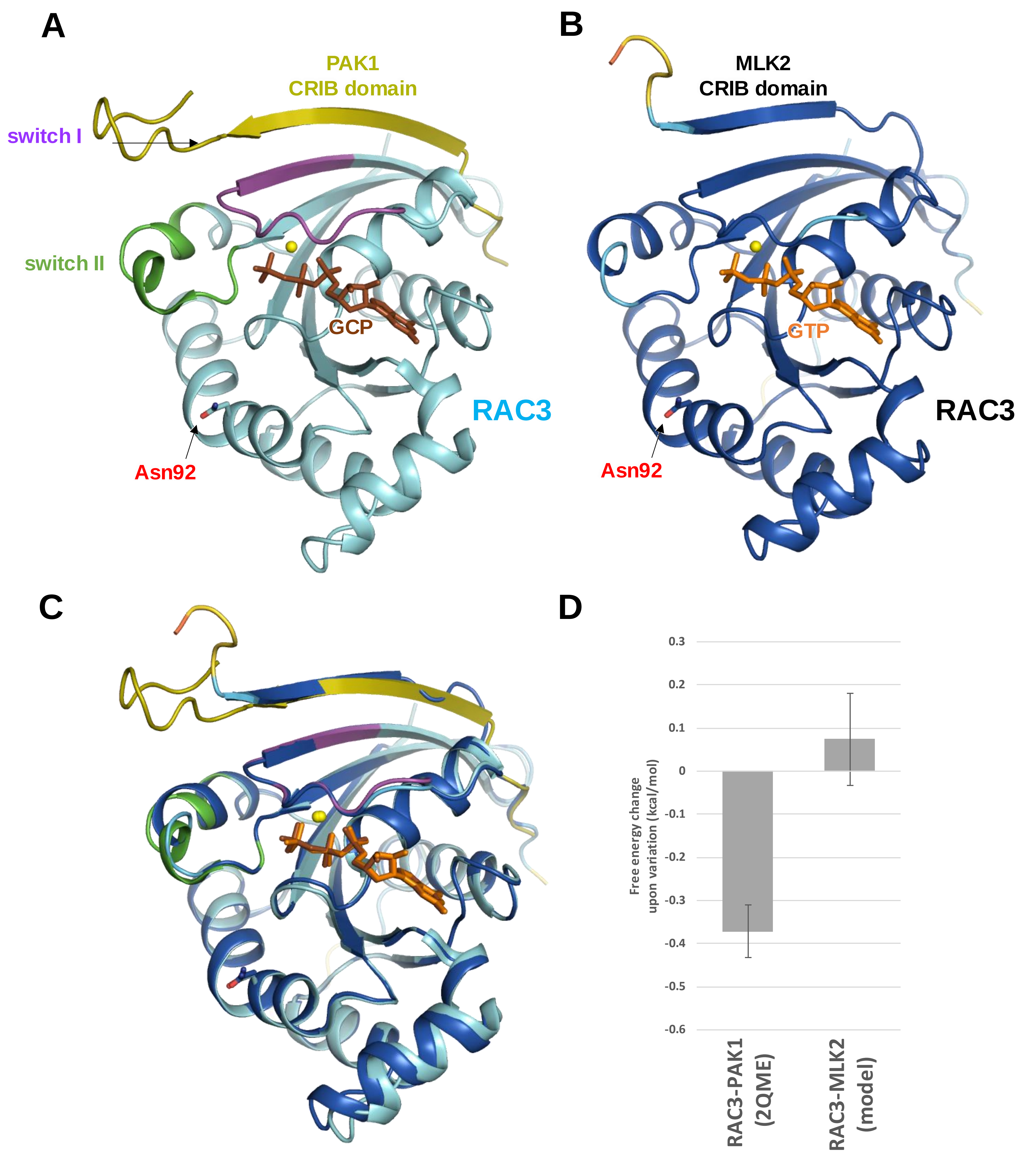

Supplementary Fig. 5. Structural overviews of the crystal structure of the RAC3-PAK1 complex (PDB: 2QME) and the AlphaFold3-predicted model of the RAC3-MLK2 complex.

(A) Crystal structure of the RAC3 (residues 1-178)-PAK1 (residues 74-97) complex. RAC3 is shown in cyan with Asn92 depicted as the stick model, while the CRIB (Cdc42- and Rac-interactive binding) domain of PAK1 is shown in olive green. The switch I (residues 30-40) and switch II (residues 59-70) regions of RAC3 are shown in magenta and green, respectively. A non-hydrolyzable analog of GTP, phosphomethylphosphonic acid-guanylate ester (GCP), and the Mg^{2+} ion are shown as brown sticks and a yellow ball, respectively. (B) AlphaFold3-predicted model of the RAC3 (residues 1-192)-MLK2 (residues 71-92) complex. Backbone structures are colored according to the confidence score (pLDDT) (see the legend of Supplementary Fig. 2). Asn92 of RAC3 with the highest confidence score (blue) is shown by the stick model. GTP and Mg^{2+} ion are shown as orange sticks and a yellow ball, respectively. (C) Superimposed structures of (A) and (B). (D) Free energy changes upon the p. N92K variation of RAC3 in the crystal structure of the RAC3-PAK1 complex and the model of the RAC3-MLK2 complex, respectively.
