# Supplementary material for: A p.N92K variant of the GTPase RAC3 disrupts cortical neuron migration and axon elongation
Source: J Biol Chem. 2025 Feb 25;301(4):108346. doi: 10.1016/j.jbc.2025.108346 (PMC11968283; doi:10.1016/j.jbc.2025.108346)
Supplement: Supplementary 6 [file mmc6.pdf]

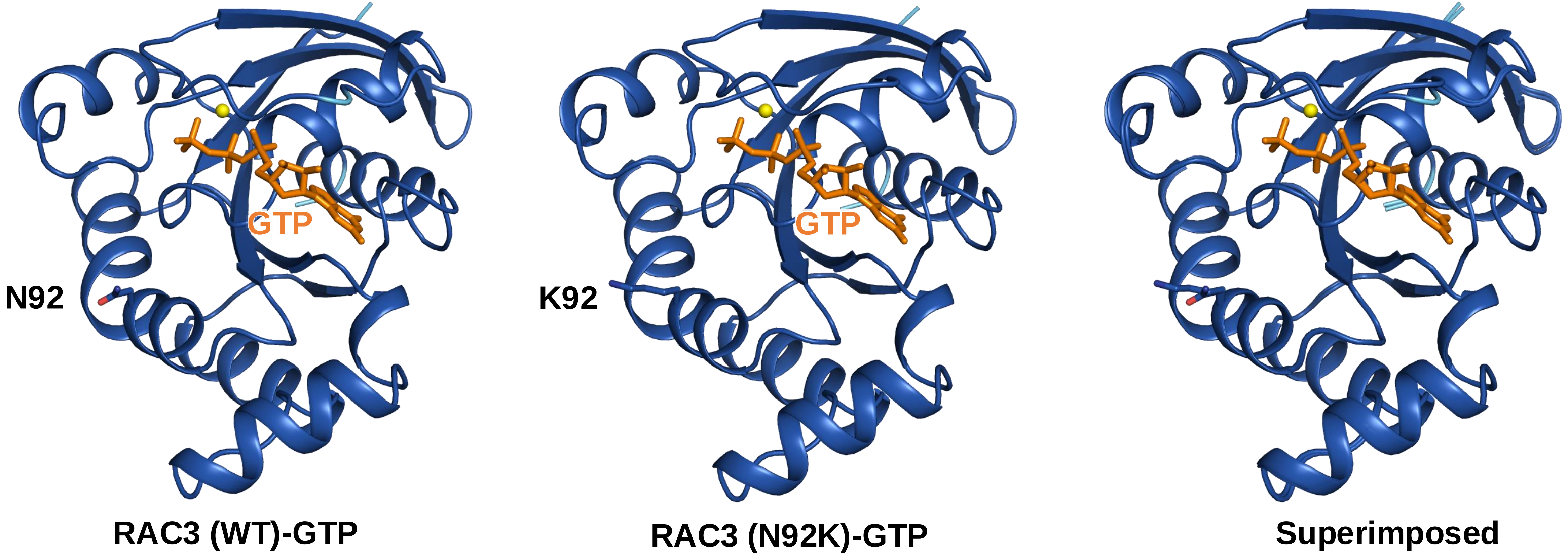

Suppleentary Fig. S6

AlphaFold3 prediction models of RAC3 (WT) (left), RAC3 (N92K) (middle), and their superimposed structures (right). Side chains of Asn92 and Lys92 are shown as stick models. Backbone structures are colored according to the confidence score (pLDDT) (see the legend of Supplementary Fig. 2). GTP and the Mg^{2+} ion are depicted as orange sticks and a yellow ball, respectively.
