# Supplementary material for: A p.N92K variant of the GTPase RAC3 disrupts cortical neuron migration and axon elongation
Source: J Biol Chem. 2025 Feb 25;301(4):108346. doi: 10.1016/j.jbc.2025.108346 (PMC11968283; doi:10.1016/j.jbc.2025.108346)
Supplement: Supplementary 7 [file mmc7.pdf]

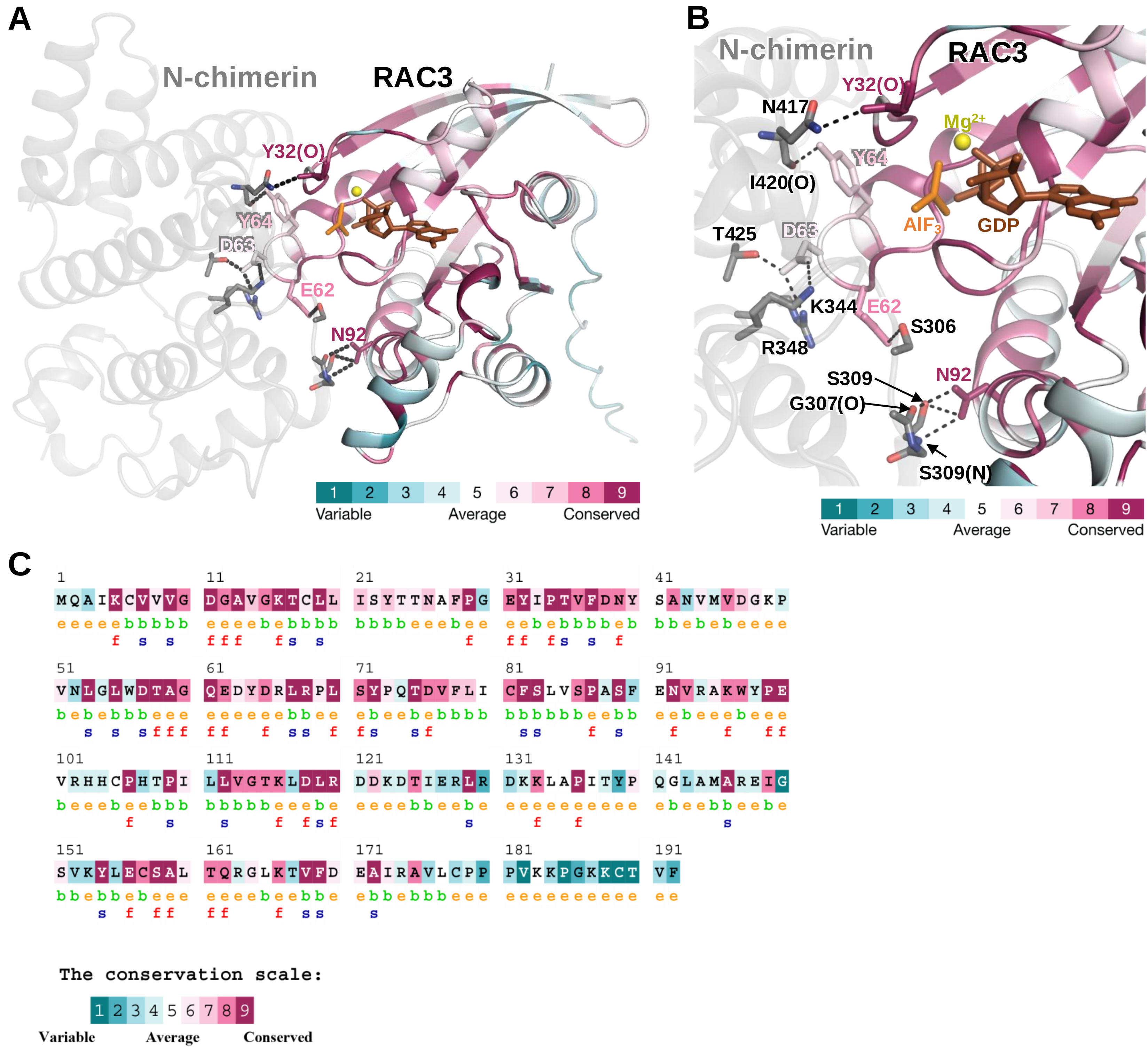

Suppleentary Fig. S7. A ConSurf analysis of the Alphafold2 prediction model of the RAC3-N-chimerin complex.

(A) The RAC3-N-chimerin complex is shown as a cartoon model, with residues involved in the binding between RAC3 and N-chimerin shown as stick models. The structure of RAC3 is colored according to conservation grades based on the sequence similarity between homologous proteins of RAC3 (RAC1-3, CDC42, RhoA, C, G, J, Q, and U) using the ConSurf program. (B) Close-up view of the interaction site between RAC3 and N-chimerin is shown. Hydrogen bonds and salt bridges are indicated by dotted lines. Labels (O) and (N) indicate the backbone oxygen and nitrogen atoms, respectively. (C) Amino acid sequence of human RAC3 with conservation grades according to the ConSurf algorithm. Residues are annotated as 'e' (exposed) or 'b' (buried). In addition, 'f' indicates predicted functional residues (highly conserved and exposed), while 's' indicates predicted structural residues (highly conserved and buried).
